# Genetic and Phenotypic Characterization of a *Salmonella enterica* serovar Enteritidis Emerging Strain with Superior Intra-macrophage Replication Phenotype

**DOI:** 10.3389/fmicb.2016.01468

**Published:** 2016-09-16

**Authors:** Inna Shomer, Alon Avisar, Prerak Desai, Shalhevet Azriel, Gill Smollan, Natasha Belausov, Nathan Keller, Daniel Glikman, Yasmin Maor, Avi Peretz, Michael McClelland, Galia Rahav, Ohad Gal-Mor

**Affiliations:** ^1^Infectious Diseases Research Laboratory, Sheba Medical CenterTel-Hashomer, Israel; ^2^Faculty of Medicine in the Galilee, Bar-Ilan UniversitySafed, Israel; ^3^Department of Microbiology and Molecular Genetics, University of California at Irvine, IrvineCA, USA; ^4^Microbiology Laboratory, Sheba Medical CenterTel Hashomer, Israel; ^5^The Department of Health Management, Ariel UniversityAriel, Israel; ^6^Galilee Medical CenterNahariya, Israel; ^7^Wolfson Medical CentreHolon, Israel; ^8^Sackler Faculty of Medicine, Tel Aviv UniversityTel-Aviv, Israel; ^9^Baruch Padeh Medical CenterPoriya, Israel; ^10^Department of Clinical Microbiology and Immunology, Sackler Faculty of Medicine, Tel Aviv UniversityTel-Aviv, Israel

**Keywords:** *Salmonella enterica*, Enteritidis, outbreak, food born infection, virulence, pathogenicity, intracellular replication, gastroenteritis

## Abstract

*Salmonella enterica* serovar Enteritidis (*S*. Enteritidis) is one of the ubiquitous *Salmonella* serovars worldwide and a major cause of food-born outbreaks, which are often associated with poultry and poultry derivatives. Here we report a nation-wide *S*. Enteritidis clonal outbreak that occurred in Israel during the last third of 2015. Pulsed field gel electrophoresis and whole genome sequencing identified genetically related strains that were circulating in Israel as early as 2008. Global comparison linked this outbreak strain to several clinical and marine environmental isolates that were previously isolated in California and Canada, indicating that similar strains are prevalent outside of Israel. Phenotypic comparison between the 2015 outbreak strain and other clinical and reference *S*. Enteritidis strains showed only limited intra-serovar phenotypic variation in growth in rich medium, invasion into Caco-2 cells, uptake by J774.1A macrophages, and host cell cytotoxicity. In contrast, significant phenotypic variation was shown among different *S*. Enteritidis isolates when biofilm-formation, motility, invasion into HeLa cells and uptake by THP-1 human macrophages were studied. Interestingly, the 2015 outbreak clone was found to possess superior intra-macrophage replication ability within both murine and human macrophages in comparison to the other *S*. Enteritidis strains studied. This phenotype is likely to play a role in the virulence and host-pathogen interactions of this emerging clone.

## Introduction

The bacterial species *Salmonella enterica* is a Gram-negative, facultative intracellular human and animal pathogen responsible for a significant public health concern worldwide (Galanis et al., 2006). *S*. *enterica* is a highly diverse species that includes more than 2,600 different serovars (biotypes). These serovars are classified into six taxonomic subspecies with high genetic similarity (Edwards et al., 2002). Various (∼1500) *Salmonella* serovars belonging to Subspecies I are known to be associated with mammals and avian hosts and accountable for the majority (∼99%) of the *Salmonella* infections in humans. Non-typhoidal *Salmonella* serovars (NTS) normally cause in humans a self-limited gastroenteritis associated with acute intestinal inflammation and diarrhea (Zhang et al., 2003). *Salmonella* infection is still one of the most common causes of food-borne illnesses and a leading cause of diarrheal diseases in developed and developing countries. 93.8 million cases of gastroenteritis due to NTS infection are estimated annually, resulting in approximately 155,000 deaths (Majowicz et al., 2010). In the USA alone, one million domestically acquired *Salmonella*-associated foodborne illnesses are reported each year, with more than 19,000 hospitalizations and at least 378 deaths, more than any other bacterial pathogen (Scallan et al., 2011).

*Salmonella enterica* serovar Enteritidis (*S*. Enteritidis) is one of the ubiquitous *Salmonella* serovars worldwide. In the last decade, an increase in the number of *S*. Enteritidis infections has been reported in the USA (Chai et al., 2012), Canada (Nesbitt et al., 2012), and the European Union (ECDC, 2009), such that *S*. Enteritidis has become the most frequently reported *Salmonella* serovar. Investigations of outbreaks as well as sporadic cases were able to repeatedly link *S*. Enteritidis infections with poultry and poultry derivatives. It has been estimated that approximately 64% of *S*. Enteritidis clinical cases are due to contaminated eggs and 18% are due to poultry products (Velge et al., 2005; Gould et al., 2013). *S*. Enteritidis is therefore a major cause of outbreaks responsible for 32% of all salmonellosis outbreaks with a confirmed serotype in the USA (Gould et al., 2013).

Like other *S. enterica* serovars, *S*. Enteritidis pathogenicity is largely determined by the ability of this pathogen to invade non-phagocytic cells and survive (often grow) within professional phagocytic cells, like macrophages and dendritic cells [reviewed in (Gal-Mor et al., 2014)]. While in most cases, NTS infections in humans cause gastroenteritis, occasionally NTS infections can result in an extra-intestinal invasive disease, with clinical manifestations of bacteremia and focal systemic infections. Recently we showed that while the average invasive (systemic) rate of NTS infections in Israel is 3.6%, *S*. Enteritidis has a significantly higher chance to cause an invasive disease (Marzel et al., 2014), thus increasing the public health concern associated with this particular serovar.

Here we report a recent *S*. Enteritidis national outbreak, which occurred in Israel during the last third of 2015. Whole genome sequencing (WGS) and comparison with global isolates of *S*. Enteritidis revealed a close genetic similarity with a domestic 2008 clinical isolate, but also with several isolates that were obtained from marine origins in the west coast of the USA and Canada, indicating that genetically related strains are circulating outside of Israel. A broad physiological and virulence-associated phenotypic characterization of this outbreak strain demonstrated a prominent ability to replicate within macrophages that is likely to affect the virulence potential of this strain.

## Materials and Methods

### Bacterial Strains and Growth Conditions

*Salmonella* Enteritidis strains used in this study are listed in **Table 1**. *Salmonella* cultures were routinely maintained in liquid or agar Lennox Luria-Bertani (LB; BD Difco) and were grown at 37°C in a shaker incubator rotating at 250 rpm.

**Table 1:** Bacterial strains and clinical isolates used in the study.

Strain	Isolation date	Source	PFGE outbreak pattern (Y/N)	Obtained from
*S*. Enteritidis 24352	11.06.2008	blood	N	Sheba Medical Center
*S*. Enteritidis 37007^∗^	01.09.2008	blood	Y	Sheba Medical Center
*S*. Enteritidis 49906	27.11.2008	blood	N	Sheba Medical Center
*S*. Enteritidis 51765	11.12.2008	blood	N	Sheba Medical Center
*S*. Enteritidis 44519	24.09.2010	blood	N	Sheba Medical Center
*S*. Enteritidis 43527	30.10.2011	blood	N	Sheba Medical Center
*S*. Enteritidis 44325	06.11.2011	blood	N	Sheba Medical Center
*S*. Enteritidis 130135377	24.08.2013	blood	N	Sheba Medical Center
*S*. Enteritidis 130138950	20.09.2013	blood	N	Sheba Medical Center
*S*. Enteritidis 130139439	24.09.2013	blood	N	Sheba Medical Center
*S*. Enteritidis 140113933	01.04.2014	blood	N	Sheba Medical Center
*S*. Enteritidis 14011035	07.01.2015	blood	N	Sheba Medical Center
*S*. Enteritidis 150118463^∗^	24.04.2015	blood	Y	Sheba Medical Center
*S*. Enteritidis 150315155^†^	15.06.2015	urine	N	Sheba Medical Center
*S*. Enteritidis 150131087^†^	19.07.2015	blood	N	Sheba Medical Center
*S*. Enteritidis 150136120	18.08.2015	blood	Y	Sheba Medical Center
*S*. Enteritidis 150145602	16.10.2015	blood	Y	Sheba Medical Center
*S*. Enteritidis 150146098	20.10.2015	blood	Y	Sheba Medical Center
*S*. Enteritidis 150148241	03.11.2015	blood	Y	Sheba Medical Center
*S*. Enteritidis 150149413	11.11.2015	blood	Y	Sheba Medical Center
*S*. Enteritidis 150149475^∗†^	11.11.2015	blood	Y	Sheba Medical Center
*S*. Enteritidis 150150208	16.11.2015	blood	Y	Sheba Medical Center
*S*. Enteritidis 150152213	29.11.2015	blood	Y	Sheba Medical Center
*S*. Enteritidis 15047444	30.11.2015	blood	N	Sheba Medical Center
*S*. Enteritidis 150153497^†^	08.12.2015	blood	N	Sheba Medical Center
*S*. Enteritidis PT4^†^	N/A	N/A	N/A	SGSC4901 reference strain
*S*. Enteritidis 665110208^†^	04.08.07	stool	N/A	2007 outbreak strain

*^∗^strain was sequenced. ^†^strains used in phenotypic assays. SGSC, *Salmonella* genetic Stock Center, the University of Calgary.*

### Pulsed-Field Gel Electrophoresis

Pulsed-field gel electrophoresis analysis was carried out as was previously described (Gal-Mor et al., 2012) and according to the CDC PulseNet protocol standardized for *S. enterica* (Ribot et al., 2006). *XbaI* and *SpeI* digested *Salmonella* DNA embedded in agarose plugs were analyzed by PFGE at 14°C in a CHEF DR III system (Bio-Rad) using the following protocol: Voltage, 6 V/cm for 19 h; initial pulse, 2 s; final pulse, 54 s; angle, 120°; in a 0.5 × Tris-borate-EDTA buffer. *S.* Braenderup strain H9812 was used as a molecular standard for this analysis.

### Motility Assay

*Salmonella* cultures grown for overnight in LB broth at 37°C were used for motility measurements. 10 μl from each culture were placed in the center of LB 0.3% agar plates and incubated for 5 h at 37°C without being inverted. Motility halo radius was measured with a ruler.

### Biofilm Formation

Overnight cultures grown in LB (to OD_600_ 4.5) were diluted 1:100 into fresh medium and 150 μl were added into cell culture treated 96-well microplates (Greiner bio-one). Negative control included LB broth only. The plates were incubated at 28°C for 96 h. Planktonic cells were discarded and attached cells were fixed for 2 h at 60°C. Fixed bacteria were stained with 150 μl of 0.1% Crystal Violet for 10 min at room temperature. The plates were washed with phosphate buffered saline (PBS) and the dye bound to the adherent bacteria was re-solubilized with 150 μl of 33% acetic acid. The optical density of each tube was measured at 560 nm.

### Tissue Cultures

All cell lines were purchased from the American Type Culture Collection (ATCC) and were cultured at 37°C in a humidified atmosphere with 5% CO_2_. The human colonic adenocarcinoma Caco-2 cell line was grown in Dulbecco’s modified Eagle medium (DMEM)–F-12 medium (Biological Industries, Israel) supplemented with 20% fetal bovine serum (FBS) and 2 mM L-glutamine. The murine macrophage-like J774.1A and the human epithelial HeLa cells were both maintained in a high-glucose (4.5 g/liter) DMEM (Biological Industries, Israel) supplemented with 10% heat-inactivated FBS, 1 mM pyruvate, and 2 mM L-glutamine. The human myelogenous leukemia THP-1 cell line was cultured in a RMPI 1640 (Biological Industries, Israel) complemented with 2-mercaptoethanol to a final concentration of 0.05 mM, 2 mM L-glutamine, 1mM pyruvate, 1% MEM non-essential amino acids, and 20% FBS.

### Adhesion, Invasion, and Intracellular Replication Assays

Eighteen hours prior to bacterial infection, epithelial cells, and macrophages were seeded at 5 × 10^4^ and 2.5 × 10^5^ cells/ml, respectively, in a 24-well tissue culture dish. Experiments were carried out using the gentamicin protection assay as previously described (Elhadad et al., 2015). Epithelial cells were infected at a multiplicity of infection (MOI) of ∼1:50 with late logarithmic phase *Salmonella* cultures and macrophages were infected at an MOI of ∼1:10 with cultures that were grown overnight to the stationary phase. Immediately after the infection, cells were centrifuged for 5 min at 500 rpm at room temperature. At the indicated time points post infection (p.i.), cells were washed three times with phosphate-buffered saline (PBS) and extracted using lysis buffer (containing 0.1% SDS, 1% Triton X-100 in PBS). Following host cells lysis, serial dilutions of the infected cell lysate were plated onto LB agar plates and incubated at 37°C for bacterial enumeration. *Salmonella* invasion was calculated by the number of intracellular *Salmonella* CFUs at 2 h p.i. divided by the infecting inoculum, while intracellular replication was determined by the number of intracellular *Salmonella* CFUs at 24 h p.i. divided by the number of invading *Salmonella* (as determined at 2 h p.i).

Adhesion was determined as was recently described (Azriel et al., 2016) using cytochalasin D (Sigma-Aldrich), which inhibits actin cytoskeleton rearrangement and bacterial cell invasion in an actin-dependent manner. Host cells were incubated with 1 μg/ml cytochalasin D 1 h before the infection. Then, *Salmonella* cultures were added and allowed to adhere the cells for 30 min in the presence of 1 μg/ml cytochalasin D. Infected cells were washed four times with PBS and harvested using lysis buffer (as above). Adherent *Salmonella* were estimated by the number of bacterial CFUs found in association with host cells at 30 min post infection (p.i.) divided by the CFU of the infecting inoculum.

### Cytotoxicity Assay

J774A.1 cells were seeded at 2.5 × 10^5^ cells/ml in a 24-well tissue culture dish and infected with *Salmonella* cultures grown to stationary phase at MOI ∼ 1:10. Cell supernatants were collected 4.5 and 20 h p.i. and analyzed for lactate dehydrogenase (LDH) release using the CytoTox 96 Non-Radioactive Cytotoxicity Assay (Promega) according to manufactures instructions. Briefly, 50 μl of the CytoTox 96 reagent were added to 50 μl cells supernatant and incubated for 15 min at 37°C. Stop solution (50 μl) was added and the absorbance was recorded at 490 nm. Uninfected cells that were lysed by the Lysis Solution (0.8% TritonX-100) were used as positive control and medium collected from uninfected cells was used as negative control. Relative cytotoxicity was calculated as the proportion between LDH release from the infected cells (Experimental LDH Release) and supernatant LDH from the positive control (maximum LDH release) that was set to 100%.

### Whole Genome Sequencing and Bioinformatics

Whole genome shotgun assemblies for *S*. Enteritidis isolates 150149475, 37007, and 150118463 were generated at the Technion Genome Center (Haifa, Israel) using MiSeq platform and 500 cycles of Illumina’s paired end chemistry to a depth of 1191, 382, and 337-fold coverage, respectively. *De novo* assembly was done using CLC-Genomics Workbench 6.05 (CLC-bio). All of these whole Genome Shotgun projects have been deposited at DDBJ/ENA/GenBank under the accession numbers listed in **Table 2**. All publically available *Salmonella* genomes were obtained from PATRIC website^1^. Pairwise alignments of *S*. Enteritidis P125109 (as a reference) with each one of the strains was generated using the Mauve tool (Rissman et al., 2009). The pairwise alignments were analyzed using custom scripts to create a pseudomultiple alignment, where putative orthologous bases to each *S.* Enteritidis P125109 position were determined. This alignment was used to determine a SNP matrix using custom scripts. Whenever raw reads were available for isolates, reads were aligned to the *S.* Enteritidis P125109 genome using Bowtie-2 (Langmead and Salzberg, 2012). Variant detection was implemented in comparison with the reference strain using SAMtools (Li et al., 2009) and custom scripts. Only bases with a Phred score of >30 (0.1% error) in each read were used to call a consensus base, and consensus bases with a score of >60 (0.0001% error) were used to call variants. The resultant SNP matrix was further trimmed to remove sites that were not reliably called in 99% of the analyzed strains. Maximum likelihood trees were generated using FastTree (Liu et al., 2011) and the SNP matrix generated above.

**Table 2:** Sequencing and assembly parameters of the three *S*. Enteritidis sequenced isolates.

Isolate	Coverage (×fold)	Number of reads (M)	Number of contigs	N_50_ (bp)	Genome size (Mb)	Number of predicted ORFs	Accession number
150149475	1191	11.58	269	489,802	4.78	4701	MAXX00000000
37007	382	7.21	43	477,839	4.76	4776	MAXY00000000
150118463	337	6.39	56	284,254	4.75	4725	MAXZ00000000

### Statistics

ANOVA with Dunnett’s Multiple Comparison Test was used to determine differences between data sets. *Z*-test was used to determined statistical significant between proportions. *P* < 0.05 was considered statistically significant.

### Ethics

The study was approved by the Institutional Review Board of the Sheba Medical Center (approval number 2915-16-SMC). Clinical isolates obtained from patients were anonymized, processed using isolate number only and were not associated with any identifying details.

## Results and Discussion

### The Emergence of *S*. Enteritidis in Israel during 2015

**FIGURE 1:**
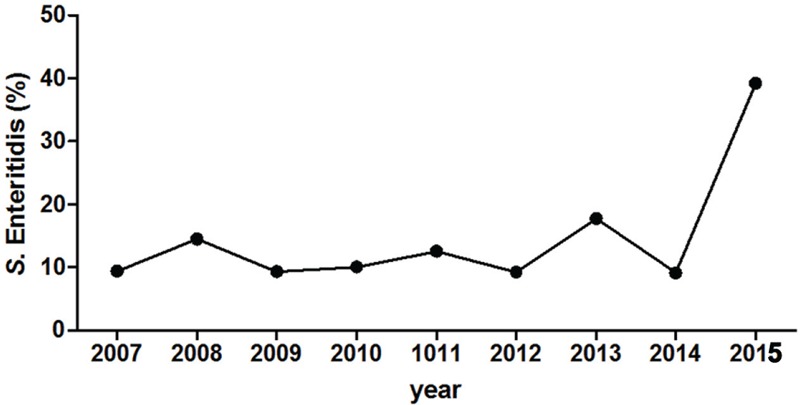
***Salmonella enterica* serovar Enteritidis emergence in Israel during 2015.** The proportion (in %) of *S*. Enteritis from all *Salmonella enterica* serovars is shown for years 2007–2015. Pooled data include the serotype of 638 *Salmonella* isolates that were collected from four Israeli medical centers (Galilee, Poriya, Wolfson, and Sheba), located in the north and the center of the country. *Salmonella* serotyping was determined at the National *Salmonella* Reference Center according to the White-Kauffmann-Le Minor scheme using O- and H-antigen specific sera.

The national prevalence of *S*. Enteritidis in Israel was rather stable from the mid-1990s until recently, accountable for about 20% of all *Salmonella* infections in humans (Gal-Mor et al., 2010; Marzel et al., 2014). Nonetheless, starting in August 2015, multiple clinical laboratories across the country have reported a sharp increase in the prevalence of *S*. Enteritidis. Integrated data from four medical centers located in the north (Galilee and Poriya) and in the center (Wolfson and Sheba) of Israel indicated a fourfold increase (*p* < 0.05) in *S*. Enteritidis infections during 2015 compared with the previous years 2007 to 2014 (**Figure 1**). These results are also in agreement with data reported to the Israel Center for Disease Control (ICDC) during October–December 2015 by other sentinel laboratories. Most of these 2015 *S*. Enteritidis isolates were susceptible to the tested antibiotics chloramphenicol, ceftriaxone, ampicillin, trimethoprim/sulfamethoxazole, ofloxacin, and ciprofloxacin. These data suggested an abnormal increase in the prevalence of this serovar in humans at the national level.

**FIGURE 2:**
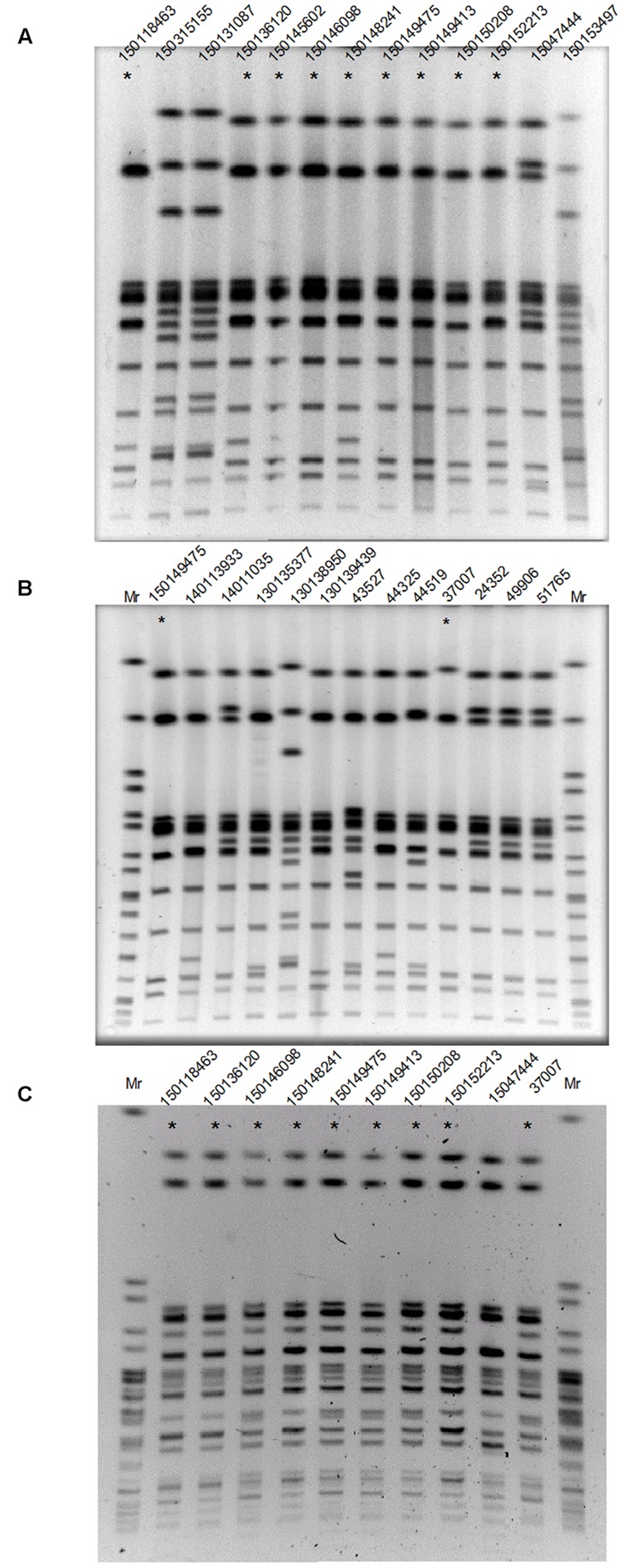
**The 2015 *S*. Enteritidis emergence is attributed to a clonal outbreak. (A)** Thirteen *S*. Enteritidis strains isolated during 2015 from patients who were hospitalized in the Sheba Medical Center were analyzed by PFGE (Pulsed-field gel electrophoresis) using *Xba*I endonuclease. **(B)** Twelve clinical *S*. Enteritidis strains collected in Sheba during 2008–2014 and the 2015 outbreak clone (isolate 150149475 as a reference) were analyzed by PFGE using *Xba*I endonuclease. **(C)** Strains that showed similar *Xba*I-PFGE profile (and isolate 1501047444 as a negative control) were digested with a second endonuclease, *Spe*I and analyzed by PFGE. DNA marker (Mr) was generated by digestion of *Salmonella* enterica serovar Braenderup H9812 DNA with *Xba*I **(B)** or *spe*I **(C)**. Strains showing the dominant 2015 outbreak PFGE profile are indicated with an asterisk.

To determine if the increased domestic prevalence of *S*. Enteritidis was the result of a clonal outbreak, PFGE was performed using *Xba*I endonuclease on 13 *S*. Enteritidis isolates from patients hospitalized in the Sheba Medical Center, during 2015 (**Figure 2A**) and 12 isolates from patients that were hospitalized in the previous years during 2008–2014 (**Figure 2B**). This analysis indicated that 9/13 of the 2015 *S*. Enteritidis isolates presented a similar PFGE profile that was distinct from most of the 2008–2014 isolates profiles.

Interestingly, the *Xba*I-PFGE pattern of the dominate 2015 outbreak clone was found to be similar to the clinical strain 37007, which was isolated from a patient blood sample at September 2008. To verify these results, nine isolates that showed similar *Xba*I-PFGE profile (and isolate 15047444 that showed a distinct profile during the outbreak as a negative control) were further examined using *Spe*I digest followed by PFGE (**Figure 2C**). These results confirmed genetic similarity between 8/9 of the 2015 isolates and demonstrated a clonal structure of the 2015 *S*. Enteritidis outbreak, which has occurred between August and November 2015. Furthermore, this analysis suggested a genetic similarity between the 2015 outbreak and the 2008 clinical isolate, 37007.

### Whole Genome Sequencing and Phylogenetic Analysis of the 2015 *S*. Enteritidis Outbreak Clone

Whole genome sequencing using MiSeq platform (500 cycle paired end run) was applied to determine the complete DNA sequence of a representative 2015 outbreak isolate 150149475 (from November 2015) and its related 2008 isolate, 37007. Another *S*. Enteritidis isolate with similar PFGE profile from April 2015 (isolate number 150118463) was also sequenced in order to determine its genetic relatedness to the outbreak. Assembly of the WGS reads indicated a genome size of 4.75–4.78 Mb and confirmed their serotyping as *S. enterica* serovar Enteritidis. **Table 2** shows the sequencing and the assembly parameters of these three *S*. Enteritidis genomes.

**FIGURE 3:**
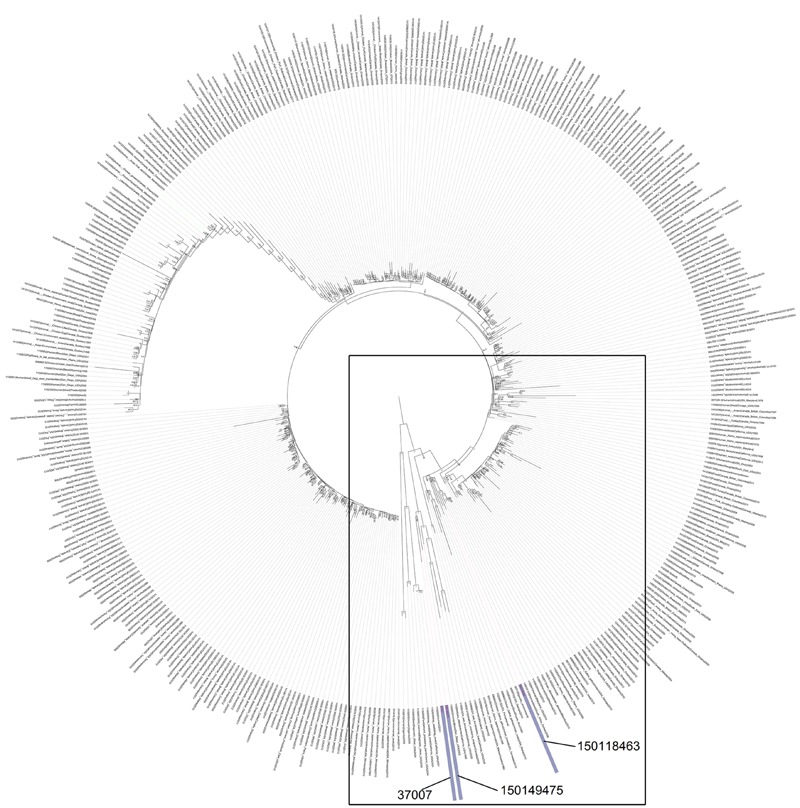
**The phylogenetic relationship of the 2015 Israeli outbreak strain with global *S*. Enteritidis isolates.** The 2015 *S*. Enteritidis outbreak strain (150149475), the 2008 clinical isolate (37007) and the April 2015 clinical isolate (150118463) were compared to *S.* Enteritidis P125109 (reference strain marked in green) in the context of 408 global *S*. Enteritidis sequenced strains. The maximum clade credibility tree was constructed based on SNPs using FastTree. The internal nodes show local support values with the Shimodaira-Hasegawa test as computed by FastTree. A zoom-in image of the boxed area, showing the close neighbors of the Israeli sequenced strains is presented in Supplementary Figure S1.

Phylogenetic analysis of the above three genomes in the context of 408 *S*. Enteritis genomes isolated from various sources around the globe, supported a close genetic similarity between isolate 150149475 and 37007, but positioned isolate 150118463 on a close, yet distinct branch (**Figure 3**), despite the fact that all three isolates shared very similar PFGE profiles. This discrepancy exhibits the disadvantage of PFGE over WGS as a molecular epidemiology tool, and indicates that the earliest strain in the outbreak that we have analyzed was isolated in August 2015 (150136120) and that the April isolated strain (150118463) is likely to be unrelated to this outbreak. Furthermore, this phylogenetic analysis showed that the 2015 *S*. Enteritidis outbreak clone is genetically related to several clinical (blood) and marine mammals (sea otter and sea lions) strains that were isolated in California USA and to a shellfish isolate from Canada (Supplementary Figure S1), indicating that closely genetic strains to the 2015 outbreak clone can be found outside from Israel.

### Physiological Characterization of the Outbreak Strain

**FIGURE 4:**
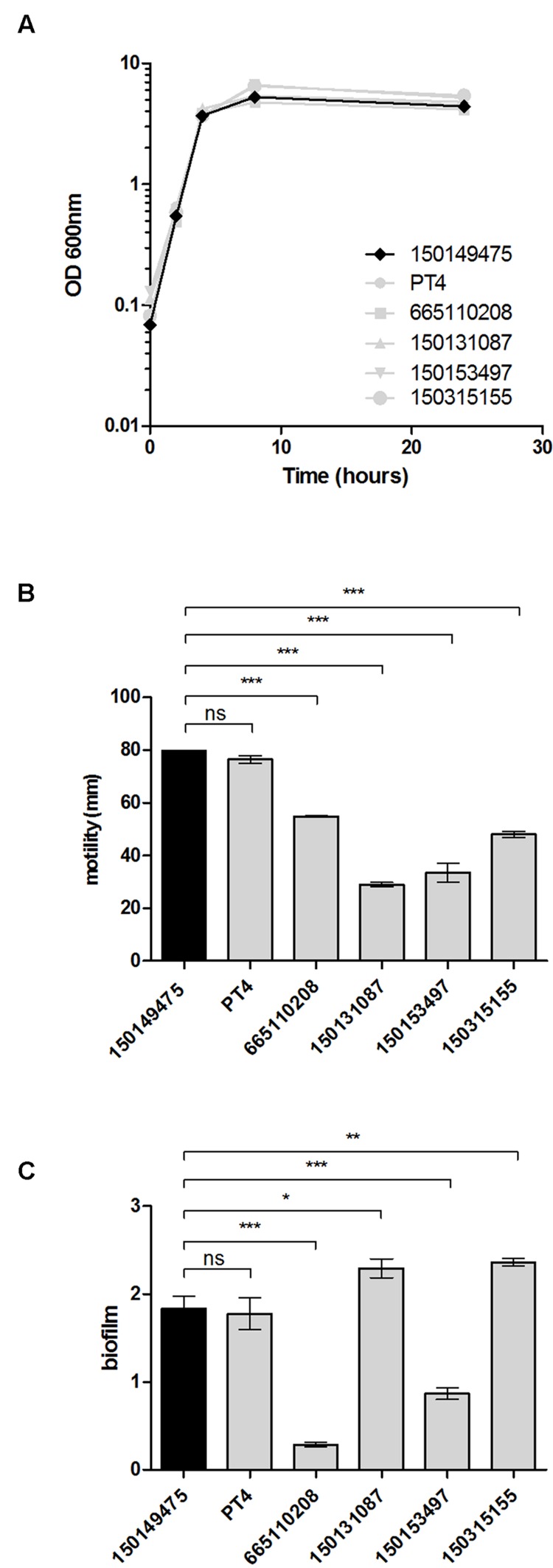
**Physiological characterization of the 2015 outbreak strain. (A)** The growth of the 2015 *S*. Enteritidis outbreak strain (150149475) was studied in rich LB medium in comparison to a reference strain (PT4) and four other *S*. Enteritidis clinical isolates (665110208, 150131087, 150153497, 150315155). Optical density at 600 nm over 24 h at 37°C is shown. **(B)** Motility of the above strains as measured on semi-solid (0.3% agar) plates following 5 h incubation at 37°C. Each bar shows the mean ± standard error of the mean (SEM) of four biological replicates. **(C)** *S*. Enteritidis strains were grown under biofilm-inducing conditions (in LB medium lacking NaCl at 28°C) for 96 h. Biofilm formation was determined using Crystal Violet staining. The bars represent the mean of six biological repeats with the SEM shown by the error bars. ANOVA with Dunnett’s Multiple Comparison Test was used to define differences between data sets. ns, not significant; ^∗^*p*<0.05; ^∗∗^*p*<0.001; ^∗∗∗^*p*<0.0001.

To characterize the 2015 outbreak clone on the phenotypic level, growth, motility, and biofilm formation of the sequenced isolate 150149475 were compared to those of a reference strain (*S*. Enteritidis PT4) and additional clinical strains, including a 2007 outbreak isolate (665110208) and three sporadic strains that were isolated in Israel prior to 2015 (3150131087, 150153497, and 150315155). Growth in rich LB medium at 37°C was very similar among all *S*. Enteritidis isolates tested, including the 2015 outbreak strain (**Figure 4A**). The 2015 outbreak strain (150149475 marked in black line or bars in **Figure 4**) and the *S*. Enteritidis reference strain (PT4) displayed similar motility (**Figure 4B**) and biofilm formation (**Figure 4C**), whereas the other strains tested were variable in these phenotypes.

### Virulence-Associated Phenotypes Characterization

**FIGURE 5:**
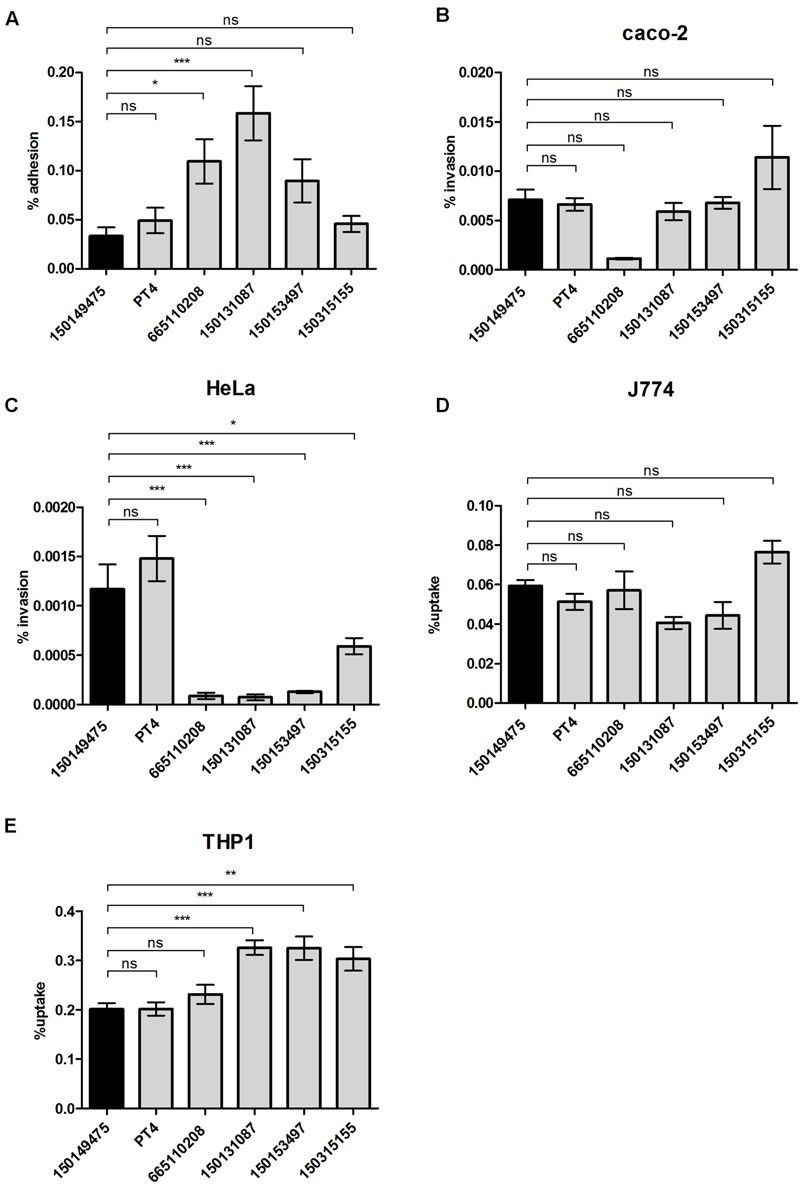
**Virulence-associated phenotypic characterization of the 2015 outbreak strain.** *S*. Enteritidis strains were grown in LB medium at 37°C and used to infect epithelial or macrophage cell-lines as specified in the “Material and Methods” Section. **(A)** Adhesion to Caco-2 cells was assayed in the presence of cytochalasin D and is presented as the percentage of cell-associated *Salmonella* from the initial *Salmonella* inoculum used to infect the cells. Invasion into Caco-2 cells **(B)** or HeLa cells **(C)** and uptake by J774A.1 murine macrophages **(D)** or THP-1 human macrophages **(E)** was studied using the gentamicin protection assay and is shown as the percentage of intracellular bacteria (CFU) recovered at 2 h p.i from the infecting inoculum. Graph bars represent the mean and SEM of 3–4 biological replicates. ns, not significant; ^∗^*p*<0.05; ^∗∗^*p*<0.001; ^∗∗∗^*p*<0.0001.

To further understand the potential virulence of the 2015 outbreak clone we studied the ability of isolate 150149475 to adhere and invade host cells. While the strains were differed in host cell adherence (**Figure 5A**), no significant difference was found between the 2015 outbreak clone and the other *S*. Enteritidis strains for invasion into human epithelial Caco-2 cell-line (**Figure 5B**) and uptake by murine J774A.1 macrophages (**Figure 5D**). Intra-serovar variation was also found in the ability to invade HeLa cells (**Figure 5C**), and in the uptake by THP-1 human macrophages (**Figure 5E**). However, in all of these virulence-associated phenotypes, the capability of the 2015 outbreak strain was similar to the one of the *S*. Enteritidis PT4 (the reference strain), with no marked characteristics that phenotypically distinct the 2015 outbreak strain from other *S*. Enteritidis isolates.

### The 2015 *S*. Enteritidis Outbreak Strain Presents an Elevated Intra-macrophages Replication

**FIGURE 6:**
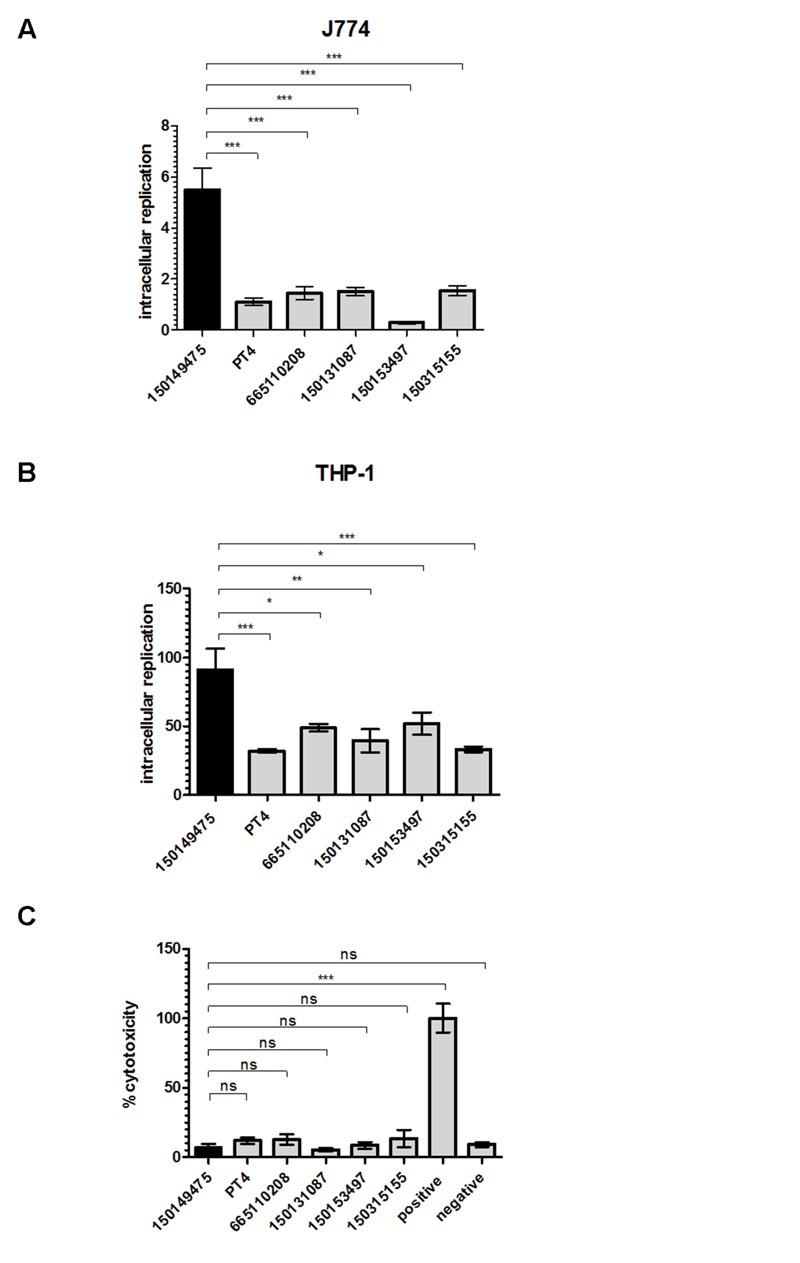
**The 2015 outbreak strain presents elevated intra-macrophage replication phenotype.** *S*. Enteritidis strains grown in LB medium to the stationary phase were used to infect macrophages in an MOI of 10. Intracellular replication of *S*. Enteritidis strains was determined in J774A.1 **(A)** or THP-1 **(B)** macrophages using the gentamicin protection assay. Bars show the mean fold-change in the number of intracellular *Salmonella* at 24 h p.i relative to 2 h p.i in 3–4 independent infections with SEM shown by the error bars. **(C)** Cytotoxicity of J774A.1 cells was tested 4.5 h post infection with *S.* Enteritidis strains. Cytotoxicity was determined by lactate dehydrogenase (LDH) release detected by colorimetric absorbance assay. Lysed uninfected cells were used as positive control and their LDH release was set to 100%. Medium collected from uninfected cells was used as negative control. Each bar shows the mean of three independent infections with SEM shown by the error bars. ns, not significant; ^∗^*p*<0.05; ^∗∗^*p*<0.001; ^∗∗∗^*p*<0.0001.

The ability of *S. enterica* to cause systemic disease is dependent upon survival and replication within macrophages (Valdez et al., 2009). *Salmonella* serovars and even different strains of the same serovar vary in their intra-macrophage survival and host cell cytotoxicity (Schwan et al., 2000; Suez et al., 2013). Therefore, we studied the intracellular replication of these strains within phagocytic cells. We found that the 2015 outbreak strain had a significantly higher intracellular replication phenotype compared to all of the other tested *S*. Enteritidis strains including PT4, both in murine J774A.1 (**Figure 6A**) and human THP-1 (**Figure 6B**) macrophages. While the average replication rate of the tested *S*. Enteritidis strains was 41 ± 9-fold over 24 h in THP-1 cells, the intra-macrophage growth of the 2015 outbreak strain was more than 90-fold. Similarly, the average intracellular growth in J774A.1 cells of *S*. Enteritidis strains was 1.19 ± 0.5-fold, while the 2015 outbreak clone growth was 5.5-fold over 24 h infection.

Previously, it has been shown that *Salmonella* spp. can induce cytotoxicity in macrophages at least in part due to a *Salmonella*-mediated apoptotic response (Chen et al., 1996; Monack et al., 1996). Hence, the observed high intra-macrophage CFU counts for isolate 150149475 could have been the result of either elevated replication or low macrophages killing by this outbreak strain. To test the possibility that the differences in intracellular replication are attributed to variation in cytotoxicity or *Salmonella*-induced apoptosis (Schwan et al., 2000), cytotoxicity assay was performed. This experiment showed low and similar J774A.1 killing by all *Salmonella* Enteritidis strains including isolate 150149475 at 4.5 h post infection (**Figure 6C**). Higher, but still similar levels of cytotoxicity between all strains were also observed at 20 h p.i. (data not shown), indicating that the elevated intracellular replication in macrophages of the 2015 outbreak isolate is not due to a particularly low cytotoxicity of its host cells. We therefore concluded from these results that in contrast to other phenotypes tested, the 2015 outbreak strain shows a distinct ability to replicate within professional phagocytic cells *in vitro*, which may suggest a higher virulence potential of this clone compared to other *S*. Enteritidis tested strains.

## Summary and Conclusion

During August to November 2015 we identified an abnormal increase in *S*. Enteritidis prevalence in Israel. PFGE analysis using two restriction enzymes confirmed the occurrence of a clonal outbreak responsible for the increase in *S*. Enteritidis infections in humans. WGS linked the 2015 outbreak strain with a clinical isolate from Israel isolated in September 2008. WGS also revealed a high genetic similarity to different clinical and environmental isolates obtained from clinical and marine origins in the west coast of USA and in Canada, indicating that the 2015 outbreak clone may not be endemic to Israel.

Phenotypic comparison between the 2015 outbreak strain with reference and other clinical *S*. Enteritidis strains showed only limited intra-serovar phenotypic variation in rich medium growth, invasion into CaCo-2 cells, uptake by J774.1A macrophage-like cells, and host cell cytotoxicity. In contrast, significant phenotypic variation occurred in biofilm-formation, motility, invasion into HeLa, and uptake by THP-1 human macrophages. Notably, the 2015 outbreak strain demonstrated significantly higher ability to replicate in both murine and human macrophages compared with reference strains. Since intra-macrophage replication plays a key role in *Salmonella* pathogenicity, it is likely that this phenotype contributes to the virulence potential of this strain.

Differences in the ability of *Salmonella* strains to replicate within macrophages could be due to varied composition or expression of numerous genes involved in *Salmonella* intra-macrophage growth. More than a decade ago, Eriksson et al. (2003) demonstrated that the transcription of more than 900 *S*. Typhimurium genes is changed during murine macrophage-like J774-A.1 cells infection and a recent RNA-seq analysis identified change in the expression of more than 1700 transcriptional start sites during *S*. Typhimurium infection of RAW 264.7 macrophage-like cells (Srikumar et al., 2015), demonstrating the genetic complexity underlying this phenotype.

Although the source of this 2015 *S*. Enteritidis outbreak have not been identified by the Israeli health authorities, future surveillance for this strain, its epidemiology and its infection outcome will provide important data, likely to shed more light on the biology and the pathogenicity of this emerging clone.

## Author Contributions

OG-M, AV, IS, and GR designed of the work. AA, PD, GS, NB, NK, DK, YM, and AP collected the data. IS, AV, SA, and OG-M performed the experiments. IS, AA, PD, SA, MM, GR, and OG-M analyzed the data. PD, MM, and OG-M wrote the manuscript.

## Conflict of Interest Statement

The authors declare that the research was conducted in the absence of any commercial or financial relationships that could be construed as a potential conflict of interest.

## Footnotes

1 http://patricbrc.org
