# Supplementary material for: Genetic and Phenotypic Characterization of a Salmonella enterica serovar Enteritidis Emerging Strain with Superior Intra-macrophage Replication Phenotype
Source: Front Microbiol. 2016 Sep 16;7:1468. doi: 10.3389/fmicb.2016.01468 (PMC5025531; doi:10.3389/fmicb.2016.01468)
Supplement: Supplementary file 1 [file Data_Sheet_1.DOCX]

Genetic and phenotypic characterization of a *Salmonella enterica* serovar Enteritidis emerging strain with superior intra-macrophage replication phenotype

Supplementary data

**Figure S1**. A zoom-in view of the tree presented in Fig. 3 showing the phylogenetic relationship of isolates 150149475, 37007 and 150118463 with their close identified neighbors. The internal nodes show local support values with the Shimodaira-Hasegawa test as computed by FastTree. Isolate number, source, place of origin and the year of isolation are indicated. The sequenced Israeli strains are marked in red.
