# Supplementary figures and images for: Genetic and Phenotypic Characterization of a Salmonella enterica serovar Enteritidis Emerging Strain with Superior Intra-macrophage Replication Phenotype
Source: Front Microbiol. 2016 Sep 16;7:1468. doi: 10.3389/fmicb.2016.01468 (PMC5025531; doi:10.3389/fmicb.2016.01468)

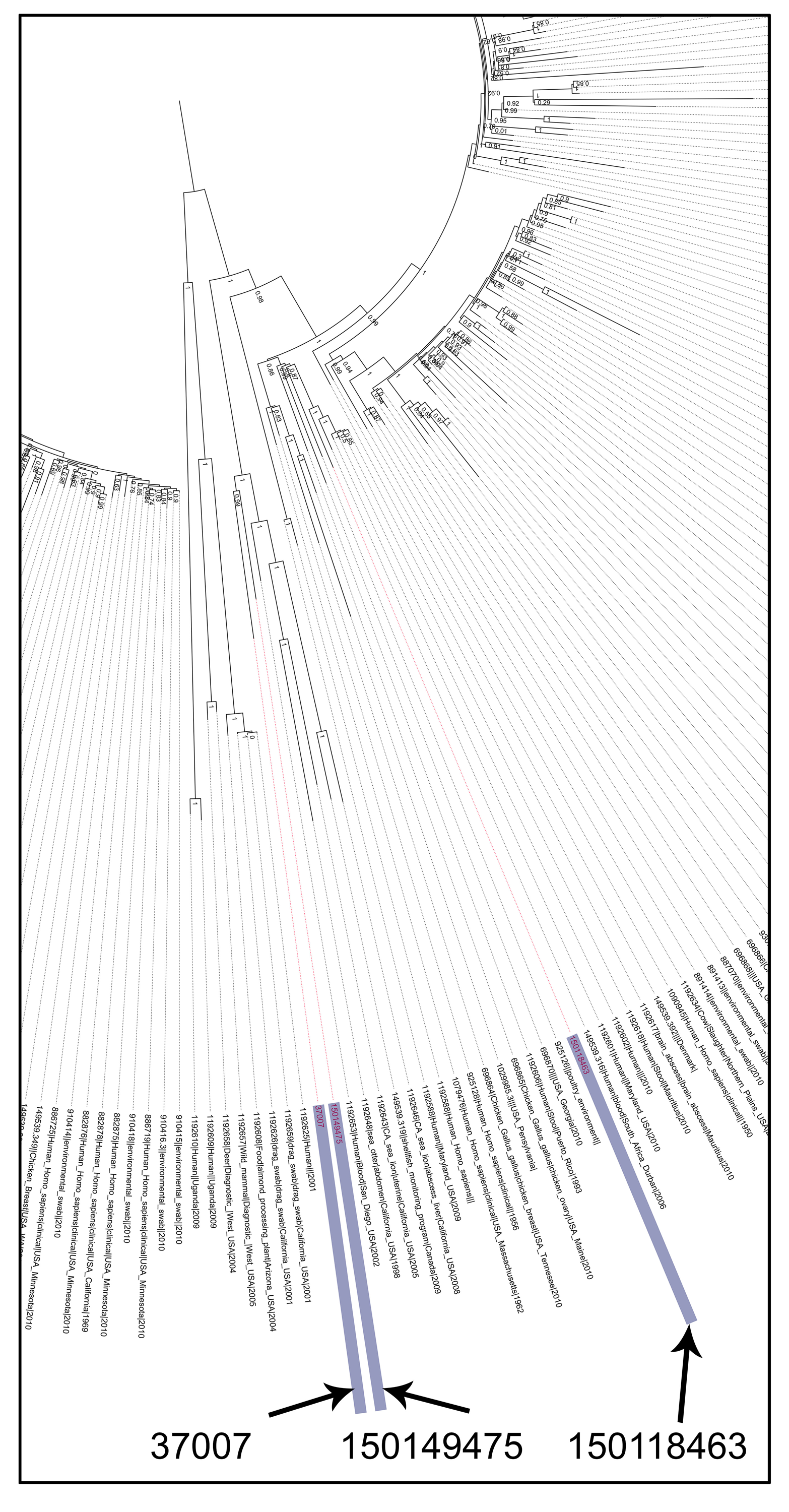

Supplement: Supplementary file 2 [file Image_1.TIF]
